# Diabetes-Mediated Toxicity Resulted in the Expression of CD80 and CD86 on Neutrophils after Delayed Wound Healing in Male Rats

**DOI:** 10.1155/2020/3592425

**Published:** 2020-07-13

**Authors:** Hossam Ebaid, Bahaa Abdel-Salam, Iftekhar Hassan, Jameel Al-Tamimi, Alli Metwalli, Ahmed Rady, Ibrahim M. Alhazza

**Affiliations:** ^1^Department of Zoology, College of Science, King Saud University, P.O. Box 2455, Riyadh 11451, Saudi Arabia; ^2^Department of Zoology, Faculty of Sciences, Minia University, El-Minia, Egypt; ^3^Department of Dairy, Faculty of Agriculture, Minia University, El-Minia, Egypt

## Abstract

**Background:**

Polymorphonuclear neutrophils (PMNs) play an essential role in the innate immune response, and their number increases after prolonged inflammatory diabetic wounds and prolonged wounds in older rats. The expression of CD80 and CD86 on PMNs confirms their participation in acquired immunity, wherein these molecules are involved in antigen presentation.

**Materials and Methods:**

We investigated CD80 and CD86 expression on PMNs by flow cytometry and analyzed the mRNA expression of neutrophil chemoattractants macrophage inflammatory protein-2 (*MIP-2*) and *MIP-1α* by real-time polymerase chain reaction (PCR) in diabetic wound, which was healed by a camel milk peptide (CMP). The animals were allocated to the following wounded groups: control, diabetic (DM), and diabetic treated with CMP (DM-CMP).

**Results:**

Alkaline phosphatase, gamma-glutamyl transpeptidase, and lactate dehydrogenase levels were elevated in DM rats but decreased in peptide-treated rats. The expression of CD80 and CD86 was significantly higher in DM rats with prolonged wounds than in control rats. The expression of both markers was restored to normal levels in diabetic rats treated with CMP. RT-PCR analysis revealed the upregulation in *MIP-2* mRNA expression in DM rats. However, neutrophil number at wounded sites of DM rats declined at day 1 after wounding as compared to that in control rats. *MIP-2* mRNA expression and neutrophil number were restored in CMP-treated diabetic rats.

**Conclusion:**

Prolonged wound stress induced toxicity in DM rats and significantly increased the expression of CD80 and CD86 on PMNs. CMP peptide ameliorated the levels of toxicity markers, CD80 and CD86, and chemoattractant molecules in diabetic rats.

## 1. Introduction

Polymorphonuclear neutrophils (PMNs) have a very short half-life (*t*^1/2^) in the circulatory system, as these cells undergo constitutive apoptosis [1]. Under special circumstances, they perform a vital role as the effector arm of the host immune defense by clearing immune complexes, phagocytosing opsonized particles, or releasing inflammatory mediators [2]. Recent investigations have considerably changed our perception about PMNs from being considered as the first-line defense against microbial infection to frontline contributors in ameliorating chronic inflammation and regulating the immune system. It is believed that these cells participate in chronic inflammatory diseases and dictate the immune response upon appropriate activation [3].

Several studies have indicated the surface expression of costimulatory molecules (CD80 and CD86) on PMNs from diversified species [4]. However, diabetic PMNs express CD80 and CD86 and participate in antigen presentation and consequently T-cell proliferation during bacterial infection. Hence, stimulated PMNs may also be engaged in immune response aside from their contribution to the innate immunity.

During the inflammatory stage of wound healing, neutrophils are responsible for microbial clearance at the site of wound infection. Further, they also contribute to antigen presentation, phagocytosis, and production of inflammatory cytokines and growth factors [5]. Hence, recruitment of appropriately functioning neutrophils is critical for the efficient removal of microbial agents to promote normal healing [6]. We have previously proved that neutrophils participate in the normal inflammatory phase of wound healing in older rats [7].

Interleukin-8 (IL-8) is an important interleukin that promotes phagocytosis following recruitment of neutrophils and macrophages at wounded site. It is also a potent promoter of angiogenese and induces a series of physiological responses in target cells that are needed for migration and phatocytosis, including upregulation in the level of intracelluar of Ca^2+^, exocytosis (e.g., histamine release), and respiratory burst.

Macrophage inflammatory protein- (MIP-) 2, also known as chemokine ligand (CXCL2), is one of the CXC chemokines that alters the recruitment and activation of neutrophils through the p38/mitogen-activated protein kinase-dependent signaling pathway via binding to its specific receptors, CXCR1 and CXCR2. This protein is synthesized in different cells such as macrophages, monocytes, epithelial cells, and hepatocytes during pathogenic invasion or tissue injury. As MIP-2 plays an important role in the regulation of neutrophil infiltration and microabscess formation, information on its functions and signaling network may generate novel ideas to cease chronic inflammation and associated complications [8].

In general, neutrophil number gradually decreases at wounded sites as healing progresses. These cells disappear in the remodeling phase when inflammation and/or bacterial load is reduced. However, we have previously noted that PMN population gradually increases in diabetic rats [9] and older rats with impaired wound healing [7]. Thus, PMNs may perform the function of antigen presentation at wounded sites after a prolonged nonefficient inflammatory phase. Here, we hypothesized that PMNs express CD80 and CD86, the antigen presentation markers, during delayed healing in diabetic rats.

Rayner et al. [10] suggested that the mixture of factors in bovine milk exerts a direct action on the cells of cutaneous wound repair to enhance both normal and compromised healing [10]. Recently, we provided evidence for the potential impact of whey protein (WP) in the treatment of immune impairment in T1D, suggesting that it serves to reverse autoimmunity by suppressing autoreactive T-cells and downregulating TNF-*α* and Fas, resulting in improved pancreatic ß-cell structure and function. WP has been shown to be able to regulate impaired wound healing normally [7]. Moreover, data provides evidence for the potential impact of WP in the upregulation of Hsp72 and Krt16 in T1D, resulting in an improved wound healing process in diabetic models [11]. In addition, postcaesarean treatment with WP promoted significant wound healing in the skin incision and had a significant wound healing potential in the uterus [12].

## 2. Materials and Methods

### 2.1. Preparation and Hydrolysis of Camel Milk Whey Proteins

Camel milk was centrifuged at 5000 × *g* for 20 min. The milk was acidified to pH 4.3, and the casein obtained was collected. The supernatant (whey protein, WP) was further saturated with ammonium sulfate. The WP was collected and dialyzed for 48 h at 4°C using a Spectra/Pro® Membrane, MWCO 6000–8000 Da. The WP was finally lyophilized using a Unitop 600 SL (Virtis Company, Gardiner, New York 12525, USA) and stored at −20°C until use [13]. A 2.5% WP solution was prepared in water at pH 7.0 using 1 mole sodium hydroxide (NaOH). Trypsin was added to WP at a ratio of 1/100 and then inactivated by warming the sample in boiling water bath for 5 min. The sample was cooled and stored in a refrigerator. Protein hydrolysis was determined by the orthophthaldialdehyde method. The resulting fractions were tested for their bioactivities. Among them, the peptide fraction 1 (CMP) was chosen for in vivo wound healing experiments [14].

### 2.2. Experimental Design and Ethical Approval

Male adult albino rats (about 6 months old) were obtained from the Department of Zoology, College of Sciences, King Saud University, Riyadh. The rats were allocated to four groups (*n* = 15). Two groups, wounded normal (CN+) and wounded diabetic (DM), were daily administered with the vehicle solution of phosphate-buffered saline (PBS; 1000 *μ*L/rat) by gastric intubation for 2 days (*n* = 5), 4 days (*n* = 5), or 7 days (*n* = 5). The third group included wounded diabetic rats that were daily treated with CMP. Rats of the third group were supplemented with CMP in a dose of 100 mg/kg of body weight via gastric intubation. The fourth group comprised wounded diabetic rats daily treated with 10 kDa of hydrolyzed WP at a dose of 100 mg/kg body weight (1000 *μ*L/rat) by gastric intubation for 2 days (*n* = 5), 4 days (*n* = 5), or 7 days (*n* = 5).

Camel milk was obtained from Majaheem camel breed from the Alazeria farm (Najd region; GPS: 300 02 47/300 02 27) in Saudi Arabia. No specific permissions were required for research activities in this farm, as the study did not involve endangered or protected species. All procedures were conducted in accordance with the standards set forth in the guidelines for the care and use of experimental animals by the Committee for the Purpose of Control and Supervision of Experiments on Animals (CPCSEA) and the National Institutes of Health (NIH). The study protocol (care and handling of experimental animals) was approved by the Animal Ethics Committee of King Saud University, Riyadh.

### 2.3. Diabetic and Wound Models

Diabetes was induced by a single peritoneal dose of freshly dissolved streptozocin (STZ Sigma-Aldrich, USA) at 50 mg/kg body weight in 0.1 mol/L citrate buffer (pH 4.5). Control group rats were injected with citrate buffer. After 14 days of STZ injection, rats with glucose level ≥ 220 mg/dL after overnight fasting were carefully chosen as diabetic rat models.

The wound models were performed at least one month after diabetes induction. After being anesthetized, the rat's back was shaved and sterilized. The wound model in this study was established as previously described [15] with some modifications. Wound was induced with blades through full thickness of the folded skin to form a 5 mm diameter circle below the shoulder of each rat. We estimated the percentage of wound area as follows: (wound area of measured wound at a given time/area of original wound on day 0) × 100.

### 2.4. Blood Samples

Blood samples were obtained in 7.5 mL heparin-coated tubes (Sarstedt; Nümbrecht, Germany) and analyzed within 2 h. Cells were tested in whole blood samples. For fluorescence-activated cell sorting (FACS) analysis of the whole blood, an erythrocyte FACS lysing solution obtained from Becton-Dickinson (Heidelberg, Germany) was diluted 1 : 10 in bidistilled water. For cytofluorometry, fluorescent-tagged (fluorescein isothiocyanate (FITC) or phycoerythrin (PE)) antibodies were used. For CD80 and CD86 detection, PMNs in the whole blood were stained with 2 *μ*g anti-CD80-FITC and CD86-PE. Cells were analyzed by the MACS Quant Analyzer (MACS) Miltenyi Biotec and Cell Quest software (Becton-Dickinson, Heidelberg, Germany). All results were expressed as percentage of positive cells in the respective gate.

### 2.5. Enzyme-Linked Immunosorbent Assay (ELISA)

The levels of IL-8 in serum samples from experimental groups were determined using the ELISA kits purchased from Abcam Systems, UK. IL-8 concentration was determined using a spectrophotometer at 450 nm wavelength according to the manufacturers' instructions.

### 2.6. Statistical Analysis

The data of wound diameter were analyzed using one-way analysis of variance (ANOVA) followed by the least significant difference (LSD) test to compare various groups.

## 3. Results

### 3.1. Analysis of Toxicity Markers

Oxidative stress is one of the principle phenomenon involved in the damage incurred by any toxicant or infection. Hence, the study of toxicity markers is vital for toxicological assessment. Alkaline phosphatase (ALP), *γ*-glutamyl transferase (GGT), and lactate dehydrogenase (LDH) levels were evaluated for this purpose.

#### 3.1.1. ALP

**Figure 1:**
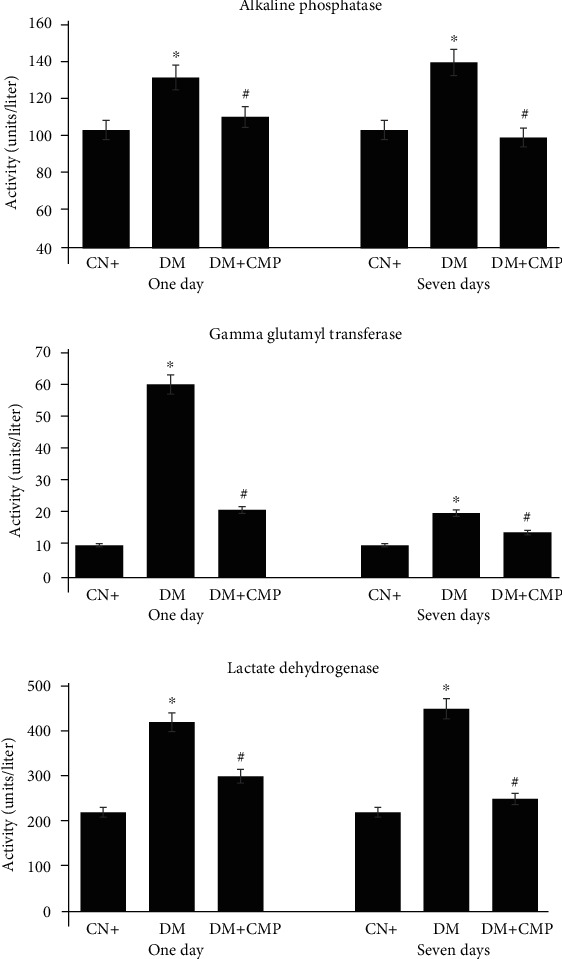
Activities of alkaline phosphatase, gamma-glutamyl transferase and lactate dehydrogenase in units per liter of samples. All values are expressed as the mean ± SEM of six different preparations. ∗ indicates significantly different from the control (group I) group. ^#^ indicates significantly different from the diabetic group II.

ALP is an important marker to assess the liver function and the extent of severity of any infection in vivo. We analyzed ALP level in the liver tissues of treated animals and found that group II animals showed a 25% elevation in ALP level. Animals from group III demonstrated a 29.22% increase in ALP level, while those from Group IV showed a decrease in ALP activity by 14.08%. The decrease in ALP activity was more evident in animals from group V (21.23% as compared to group II animals) (Figure 1).

#### 3.1.2. GGT

GGT is a reliable marker to assess the extent of infection and toxicity burden in vivo after treatment with chemicals or drugs. GGT activity significantly increased by 438.44% in group II rats, whereas group III rats showed an increase of 91.19% in GGT activity. Among the peptide-treated groups, group IV showed a decrease of 65.46%, and group V rats demonstrated a decrease of 72.97% in GGT activity as compared with group II rats (Figure 1).

#### 3.1.3. LDH

LDH activity increased in group II and III rats by 89.06% and 99.68%, respectively. However, group IV rats showed a decrease in LDH activity by 23.47%, while the activity reported for group V decreased by 39.05% as compared to that observed in group II rats (Figure 1).

### 3.2. Neutrophil Recruitment into Diabetic Wounded Tissues

**Figure 2:**
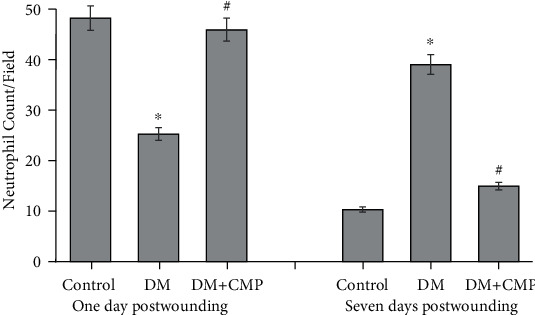
Neutrophil count during the inflammatory phase (first 24 h) and remodeling phase (after 7 days postwounding) in control, diabetic, and CMP-treated diabetic rats. Results are the average of neutrophil number per ×400 microscopic field. All values are expressed as the mean ± SEM of three different preparations. ∗ indicates significantly different from the control (group I) group. ^#^ indicates significantly different from the diabetic group.

The results shown in Figure 2 demonstrate the increase in the number of neutrophils into wounded sites. Diabetes impaired the migration of neutrophils from blood vessels to the site of inflammation after 1 day from incision. CMP was found to restore the abundance of neutrophils in diabetic rats to normal levels in wound tissues. Neutrophil migration at the site of wound in both control and CMP-treated diabetic rats significantly decreased after 7 days from incision but considerably increased in the wounded regions of diabetic rats.

### 3.3. Inflammatory Cells (PMNs) into the Dermis and Wound Closure Rate

We observed a marked decrease in the number of PMNs in the wounds of diabetic rats, consistent with healing impairment. In this group, inflammatory cell number declined in the dermal region after the first 24 h from wounding. Both neutrophil number and inflammatory cell number were restored in the dermal areas of diabetic rats treated with CMP. Wound closure rate was slower in diabetic rats than in control rats. CMP was found to restore the wound closure rate to normal level (Table 1).

### 3.4. Effect on Chemokines

#### 3.4.1. IL-8 Protein Level

IL-8 induces chemotaxis in target cells, neutrophils, and other granulocytes and facilitates migration toward the site of infection. Although IL-8 level was not significantly elevated, its concentration was higher in diabetic rats than in control and CMP-treated diabetic rats (Table 2).

#### 3.4.2. MIP-2 mRNA Expression

Gene expression of neutrophil-attracting chemokines is upregulated during impaired wound healing. RT-qPCR analysis revealed the upregulation in the mRNA expression of neutrophil chemoattractant *MIP-2* in wounded rats from all groups when neutrophil number peaked at day 1 postwounding as compared to rats from the control group. Although the mRNA expression of *MIP-2* significantly increased in diabetic and CMP-treated diabetic rats, the levels were downregulated in all rat groups after 7 days of incision (Table 2).

#### 3.4.3. MIP-1*α* mRNA Expression

The mRNA expression of macrophage chemoattractant *MIP-1α* was significantly downregulated in all diabetic and CMP-treated rats as compared to that in control rats on day 1 of incision. However, after 7 days, diabetic rats showed no difference as compared to control rats, while CMP-treated rats showed a significant upregulation in *MIP-1α* level as compared to both control and diabetic rats (Table 2).

### 3.5. Extracellular Expression Costimulatory Molecules on Blood PMNs

#### 3.5.1. Expression of CD80 on Blood PMNs

**Figure 3:**
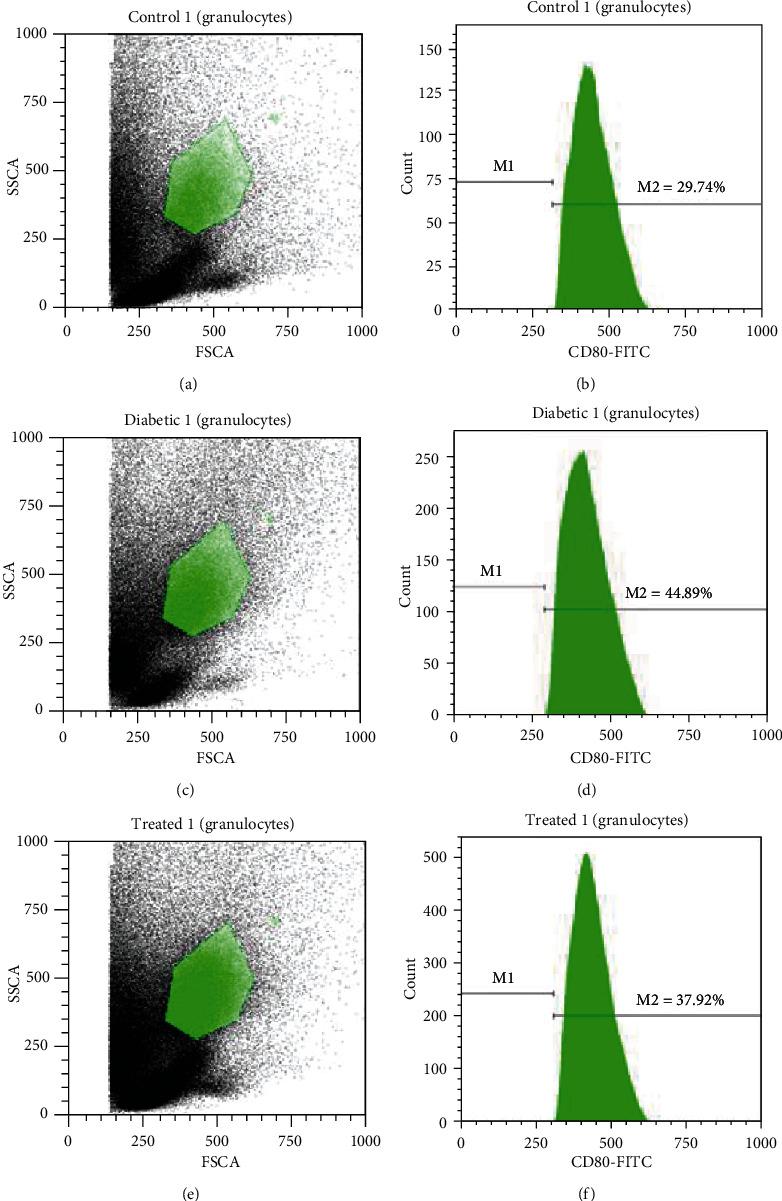
Percentages of CD80 expressed on granulocytes. (a) The granulocyte population of normal mice. (b) CD80 percentage in normal mice. (c) The granulocyte population of diabetic mice. (d) CD80 percentage in diabetic mice. (e) The granulocyte population of treated mice. (f) CD80 percentage in treated mice.

CD80 is a reliable costimulatory marker to assess the extent of antigen presentation burden on PMNs after any treatment. The expression of CD80 on the PMNs from untreated control rats was 29.74% (Figures 3(a) and 3(b)). In diabetic animals, CD80 expression significantly increased to 44.89% (Figures 3(c) and 3(d)) as compared to control rats. In contrast, CD80 expression of PMNs from rats treated with 3 kDa peptide was considerably restored to 37.92% (Figures 3(e) and 3(f)) as compared with that tested in diabetic rats.

#### 3.5.2. Expression of CD86 on Blood PMNs

**Figure 4:**
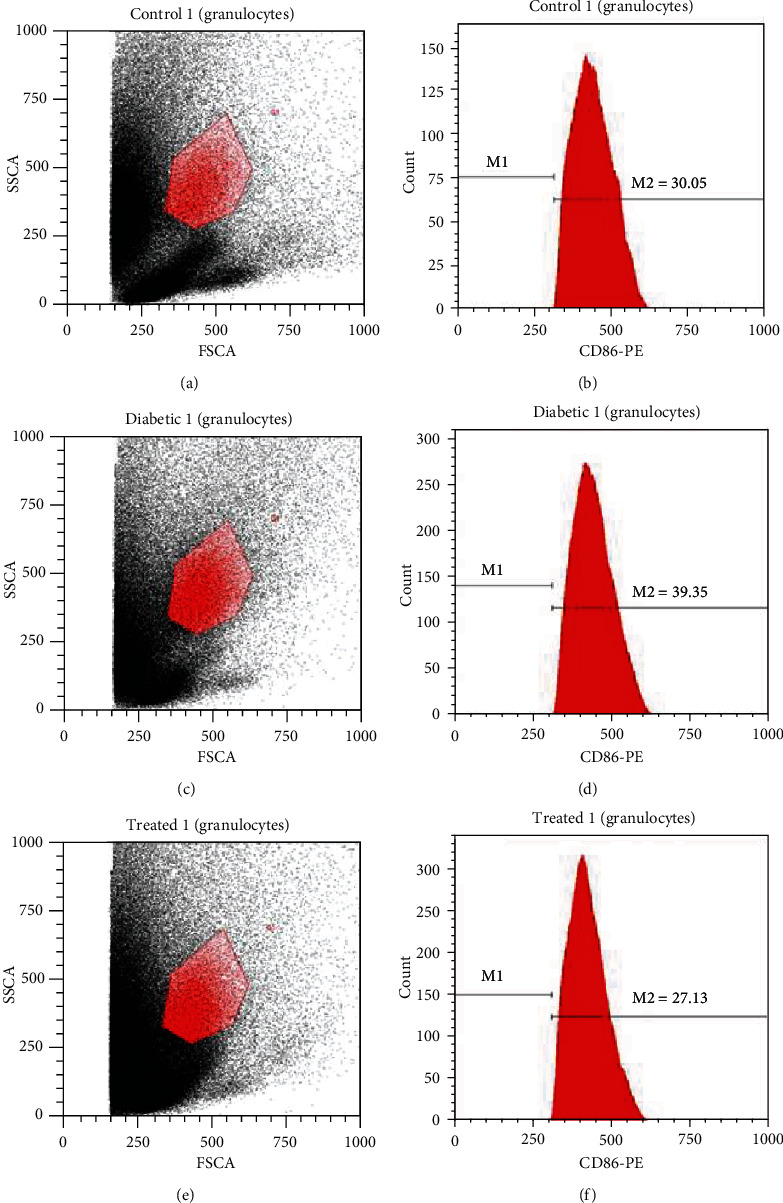
Percentages of CD86 expressed on granulocytes. (a) The granulocyte population of normal mice. (b) CD86 percentage in normal mice. (c) The granulocyte population of diabetic mice. (d) CD86 percentage in diabetic mice. (e) The granulocyte population of treated mice. (f) CD86 percentage in treated mice.

CD86 is an important costimulatory marker to assess the antigen presentation function of PMNs and to evaluate the extent of severity of any stimulated PMNs. PMNs from untreated control rats showed 30.85% CD86 expression (Figures 4(a) and 4(b)). At day 7 from wounding, the PMNs derived from wounded diabetic rats showed significantly higher expression (39.35%) of CD86 than those derived from control rats (Figures 4(c) and 4(d)). The expression of CD86 on the PMNs derived from 3KDa peptide-treated rats was significantly lower (27.13%) than that on the PMNs from untreated control rats (Figures 4(e) and 4(f)).

## 4. Discussion

A study showed that neutrophil- and macrophage-deficient mice could effectively heal wounds in a sterile environment [16]. Further, high neutrophil infiltration at late stages of healing disturbed the healing process in older rats [7]. Neutrophil depletion in diabetic C57BLKS/J-*m*+/+*Leprdb* mice resulted in faster reepithelialization [17] and depletion of circulating monocytes and macrophages in diabetic C57BL/6J-*ob/ob* mice [18] accelerated the process of healing. In the present study, we found that high numbers of neutrophils in the inflammatory phase were necessary for the normal healing of nonsterilized wounds. Delayed diabetic wound healing was consistent with the significant depletion in neutrophil recruitment into wounded regions. Moreover, the increase in neutrophil recruitment at late stages of diabetic wounds disturbed and delayed normal cellular events. To address these drastic changes in diabetic wounds, we evaluated the expression of antigen-presenting markers CD80 and CD86 on PMNs under prolonged healing stress.

The liver is a central organ in managing different functions, including digestion, xenobiotic metabolism, immune response, and detoxification. Upon liver injury, the activated Kupffer cells are the major source of MIP-2. MIP-2-recruited and MIP-2-activated neutrophils can exacerbate liver inflammation by releasing various inflammatory mediators [8]. Given the strong relationship between the immune response and liver injury, we investigated whether stress acts as a toxic insult and induces inflammatory response by evaluating major toxicity markers, including LDH, ALP, and GGT. Our findings show that these toxicity markers were significantly elevated in diabetic rats. The WP could decrease the toxic effects on one of the most metabolically active organs, the liver [19]. The elevation in levels of these markers indicates that diabetes affects functions of the liver that can consequently also compromise the immune system. On the contrary, the positive effect of the WP may be mediated through the amelioration of diabetes-associated hepatotoxicity and could consequently boost the immune system involving a complex array of cytokines and immune cells [14].

The expression of CD80 and CD86 was detected in stimulated PMNs [20], suggestive of the important role of PMNs in antigen presentation. Here, we found that the expression of these markers was significantly upregulated on diabetic PMNs after 7 days from wounding as compared with their expression on PMNs from peptide-treated and normal rats. In general, the appropriate recruitment and functions of PMNs in healthy animals are evident during inflammation [21]. We have previously reported lower number of neutrophil infiltration in the dermis at impaired wounded sites during the inflammatory stage; however, this number gradually increased and reached its peak on day 8 after wounding [22]. The increase in the number of neutrophils in diabetic wounds after 7-10 days, a prolonged inflammatory phase, may explain the upregulation in the expression of both CD80 and CD86 on PMNs. This hypothesis is confirmed from the continuous increase in the level of malondialdehyde (MDA), an oxidative stress marker, during the period of wound healing in diabetic rats [9]. Reactive oxygen species (ROS) is used by neutrophils and macrophages in the clearance of bacteria in wounded regions.

Normal wound healing was consistent with the significant increase in neutrophil recruitment into wounded regions in the early inflammatory phase. Neutrophils release active antimicrobial substances, proteinases [23], and crucial inflammatory cytokines. Whey protein and its derivative (CMP) has an ability to stimulate immune responses involving neutrophils and macrophage cytotoxicity [24]. This explains the early infiltration of neutrophils in diabetic rats supplemented with CMP. CMP acts as an anti-inflammatory by regulating the transport of NF-*κ*B from the cytosol to the nucleus [14]. The transport of NF-*κ*B from the cytosol to the nucleus initiates the production of inflammatory cytokines. Blocking the production of inflammatory cytokines has improved hemodynamic performance. Thus, in the present work, the recovery effect by CMP seemed to be mediated by the blocking of the proinflammatory cytokines through inhibition of NF-*κ*B.

Our data on neutrophil recruitment at wounded site emphasize their crucial role in bacterial clearance and highlight the expression of APC molecules on PMNs. Sharma et al. [25] found that the neutrophil population from patients with visceral leishmaniasis could induce CD80 and CD86 expression. While these neutrophils did not stimulate T-cell proliferation, their expression of programmed cell death ligand-1 was higher than that of other neutrophils, and lymphocytes from same patients showed high expression of programmed cell death ligand-1. In addition, Zheng et al. [26] found that phagocytosis of apoptotic neutrophils actively suppresses the stimulation of macrophages, thereby altering the macrophage response. Thus, the mechanism underlying neutrophil accumulation (increased recruitment and/or longer life-span) needs further investigation. Neutrophils may express CD80 and CD86 to exhibit higher association with programmed cell death ligand-1. Hence, the neutrophil-mediated transition of inflammatory macrophage M1 to anti-inflammatory macrophage M2 may occur in diabetic wounded tissues. This may explain the delay in the inflammatory stage until the expression of programmed cell death ligand-1 on neutrophils.

Much information is yet to be gathered on the precise role of each immune cell in the various stages of wound healing [27]. Here, we found that the increase in neutrophil recruitment during the wound healing remodeling phase in diabetic rats occurred simultaneously with the upregulation in CD80 and CD86 costimulatory molecule expression on PMN surfaces. This observation may highlight the role of neutrophils in impaired diabetic wound healing. Upregulation in the expression of these molecules may lead to the stimulation of T-cells and other members of the adaptive immune system that may be involved at later stages to ward-off specific pathogens. However, excessive infiltration of neutrophils in wounds at late stages may lead to chronic inflammation. Neutrophils release significant amounts of enzymes such as collagenase [28] and elastase, which can alter the levels of important healing factors such as platelet-derived growth factor (PDGF) and transforming growth factor beta (TGF-*β*) [29]. Further, the macroenvironment releasing excessive ROS may exacerbate the response and affect the healing of tissues in diabetic models [14].

PMNs are important effector cells associated with the host defense and inflammation. They have a short half-life and undergo spontaneous apoptosis in vivo as well as in cultures [6]. Recent evidence suggests that the cocultivation with cytokines may prolong the life-span of PMNs [30]. This study has clearly shown that PMNs could be induced to express CD80 and CD86 in diabetic rats. The APC molecule human leukocyte antigen- (HLA-) II and the costimulatory molecules CD80 and CD86 play important roles in T-cell proliferation, where HLA-II presents the engulfed antigen to T-cells [31]. CD80 and CD86 act as second signaling molecules involved in the stimulation of T-cells to produce the autocrine marker IL-2 without which T-cells may not undergo proliferation [32].

MIP-2 is a macrophage inflammatory protein instrumental in eliciting neutrophil response. Thus, the upregulation in MIP-2 expression may strongly induce the migration and chemotaxis of neutrophils and facilitate the secretion of inflammatory cytokines from macrophages [33]. Our results also reveal the significant increase in the number of neutrophils and inflammatory cells 7-10 days postwounding in diabetic rats. Here, both CD80 and CD86 on PMNs were found to be significantly upregulated in diabetic rats. The systemic treatment with recombinant MIP-2 may lead to a significant increase in the number of corneal PMNs and exacerbate corneal disease in resistant (cornea heals) mice [34]. On the contrary, the attenuated antibacterial ability in the inflammatory phase was associated with the induction of type I interferon and suppression of chemoattractants keratinocyte-derived chemokine and MIP-2, leading to the reduction in neutrophil infiltration to wounded sites upon secondary bacterial invasion [35]. All these phenomena may be attributed to bacterial stress as well as the toxicity which resulted from oxidative status [13]. In liver injury, neutralizing MIP-2 lowered neutrophil extravasation, and neutrophil-induced injury was reduced in a mouse model of cholestatic liver damage [36]. Many studies have demonstrated the requirement of a chemotactic signal such as MIP-2 from macrophages, hepatocytes, or even already extravasated neutrophils for the extravasation of neutrophils into the parenchyma. Hence, cellular damage and necrosis often lead to the release of damage-associated molecular patterns, resulting in the upregulation in the expression of intercellular adhesion molecule-1 on sinusoidal endothelial cells. Neutrophils are then migrated toward endothelial cells or hepatocytes through a *mechanism involving β*2 integrin macrophage antigen- (Mac-) 1-dependent adhesion [36, 37]. From the current work, it is highly speculative that a similar mechanism may occur in the skin tissue that delayed the wound healing process in diabetic rats.

## Figures and Tables

**Table 1:** Number of PMNs and inflammatory cells in the wounded region and the rate of wound closure in different rat groups.

	Number of inflammatory cells in dermal tissue (microscopic field; ×1000)		Percentage of the size of the opened area of wound after 10 days postwounding
	1 day postwounding	10 days postwounding	
Control	650 ± 43	370 ± 17	20 ± 2
Diabetes	317 ± 32∗	415 ± 22∗	32 ± 4∗
Diabetic+CMP	375 ± 37∗^#^	312 ± 15∗^#^	17 ± 3∗^#^
Diabetic+10 kDa	345 ± 29∗^#^	298 ± 21∗^#^	25 ± 1.9∗^#^

Percentage of wound closure rate after 10 days from the initial wound size at day 0. The percentage of wound area was estimated as follows: (wound area of measured wound at a given time/area of original wound on day 0) × 100. All values are expressed as the mean ± SEM of three different preparations. ∗ indicates significantly different from the control group. ^#^ indicates significantly different from the diabetic group.

**Table 2:** Concentration of the chemotactic factor, IL-8, and the RT-qPCR analysis of the relative expression of MIP-2 and MIP-1*α* mRNAs.

	Number of inflammatory cells in dermal tissue (microscopic field; ×1000)		Percentage of the size of the opened area of wound after 10 days postwounding
	1 day postwounding	10 days postwounding	
Control	650 ± 43	370 ± 17	20 ± 2
Diabetes	317 ± 32∗	415 ± 22∗	32 ± 4∗
Diabetic+CMP	375 ± 37∗^#^	312 ± 15∗^#^	17 ± 3∗^#^
Diabetic+10 kDa	345 ± 29∗^#^	298 ± 21∗^#^	25 ± 1.9∗^#^

All values are expressed as the mean ± SEM of three different preparations. ∗ indicates significantly different from the control group. ^#^ indicates significantly different from the diabetic group.

## Data Availability

The data used to support the findings of this study are included in the article.
